# American Veterinary College Commencement

**Published:** 1891-04

**Authors:** 


*American Veterinary College, Commencement.*—The 16th Annual commencement of the American Veterinary College, was held at Chickering Hall, New York, on Wednesday evening, March 8, 1891. The hall was packed to the last seat by the interested and enthusiastic friends of the graduates. The front of the stage was loaded with flowers, and Cappa's 7th Regiment Band added to the gaiety of the scene.

Prof. F. D. Weisse, M. D., President of the Board of Trustee's, called attention to the increased number of Graduates, and the greater value placed by the public on Veterinary Medicine. Dr. Liautard made the annual report to the Board of Trustees, and presented the following gentlemen, who received their degrees of D. V. S.:

Edwin Braden Ackerman, Brooklyn, N. Y.; Edgar Daniel Bachman, Easton, Pa.; E. Lyman M. Bishop, Brooklyn, N. Y.; Oscar Emil Busener, New York City.; John Matthews Buckley, New York City.; Joseph William Burby, Holyoke, Mass.; George Brinton Burchsted, Providence, R. I.; Amos Oliver Cawley, Lewisburgh, Pa.; Horace Henry Choate, Windsor, Me.; Jonathan H. Conover, Cooper Hill, N. J.; Edward Connolly, New York City.; Israel Kline Deckard, Middletown, Pa.; James Edward Delaney, New York City.; David Brush Doughty, Woodbury, L. I.; Ralph Alexander Dunn, Titusville, Pa.; Clement V. Elliott, Vincennes, Ind.; Henry Deacon Fenimore, Rancocas, N. J.; Daniel Cameron Gearhart, Lewisburgh, Pa.; George Joseph Goubeaud, Brooklyn, N. Y.; Frank Harvey, Durham, N.C.;

Anton Phillip Hess, Wheeling, W.Va.; Fred. Sterling Hewitt, Meshoppen, Pa.; Russell Chancey Hurlbert, Ava, N. Y.; John Andrew Kenny, New York City.; Hermann Kock, Brooklyn, N. Y.; William Albert Kroos, Brooklyn, N.Y.; John Payne Lowe, Jr., Paterson, N.J.; James McDonough, Montclair, N. J.; John Joseph Meehan, New York City.; George William Meyer, New York City.; Wilbnr John Murphy, New York City.; Edward James Nesbitt, Poughkeepsie, N. Y.; Edgar Odell, New York City.; Lewis Irving Palmer, West Bloomfield, N. Y.; Walter Hutson Phyfe, Delhi, N.Y.; William Erwin Smith, Sedalia; Mo.; Edgar Newton Stout, Greensburg, Ind.; Reginald Thomas, Decorah, Iowa.; La Forrest Everette Turner, Rockville Center, L. I.; Abraham Ditmars Van Siclen, Jamaica, L. I.

Prof. C. A. Doremus, M. D., announced the following prizes for the "Best general examination," a gold medal to Edward James Nesbitt, of Poughkeepsie, New York; for the "Second best general examination," a set of Standard Veterinary books, to Reginald Thomas, Decorah, Iowa; for the "Best practical examination," a gold medal, to Frank Harvey, Durham, N. C.; for "Best examination in Anatomy in the Junior Class," to Adam Wm. Ormiston; for the "Best paper read before the Medical Association, and defended, during the past year," a Case of Surgical Instruments, to Walter Hutson Phyfe, Delhi, New York. Dr. E. N. Stout gave the Valedictory for the graduating class, commencing with the conglomeration of technical terms as they appear to the student entering upon his career and coming down to practical logic at the termination, to represent their present ideas. Dr. D. B. St. Johns Rossa, M. D., gave an address eulogizing the advance and importance of Veterinary Medicine, as it is now recognized by the medical profession, together with good advice to the graduates as to their future duties and career.

The officers of the class were :

President, W. H. Phyfe; Vice-President, J. E. Delaney; Secretary, Geo. W. Meyer; Treasurer, J. McDonough; Class Historians, W. J. Murphy and J. P. Lowe; Marshall, C. V. Elliott; Commitee, I. K. Deckard, G. J. Goubeaud, E. J. Nesbitt, J. H. Conover, E. Odell, O. E. Busener, A. O. Cawley, J. S. Lamkin, T. E. Budd, J. E. Delaney, Wm. E. Smith, W. J. Murphy, W. V. Bieser, G. B. Burchsted, J. J. Meehan, J. P. Lowe, E. B. Ackerman, H. H. Choate, J. M. Buckley, E. N. Stout.

After commencement the dinner of the Alumni Association was held at the Brower House. Toasts were responded to as follows :

*American Veterinary College*, Prof. S. D. Weisse; *The Faculty*, Prof. A. Liautard; *The Medical Profession*, P. Brush; *The Veterinary Profession*, Prof. Huidekoper; *The Alumni Association*, Dr. Hoskins; *Class Toast*, Dr. W. H. Phyfe. These were followed by an entertaining class history by Dr. W. J. Murphy, of New York. Dr. Murphy said that he would deal with idioscyncrasies of the class, and he mixed facts of removal by sickness, death and other cases, with good natured raillery of members of the class, which was probably understood better by them, than by the older members present. Dr. Murphy regretted the time spent at the College in the study of Chemistry, and said that the final problem was a mixture of 70 students, plus 10 examiners, less 17 missed vocation, which results in 40 graduates, aud a residue of 13, the reaction of the latter is blue.

